# Clinical findings in patellofemoral osteoarthritis compared to individually-matched controls: a pilot study

**DOI:** 10.1136/bmjsem-2020-000877

**Published:** 2020-12-09

**Authors:** Erin M Macri, Kay M Crossley, Harvi F Hart, Agnes G d’Entremont, Bruce B Forster, Charles R Ratzlaff, David R Wilson, Karim M Khan

**Affiliations:** 1 Department of Family Practice, The University of British Columbia, Vancouver, Canada; 2 Department of General Practice; Department of Orthopaedics and Sport Medicine, Erasmus University Medical Centre, Rotterdam, The Netherlands; 3 La Trobe Sport and Exercise Medicine Research Centre, La Trobe University, Melbourne, Australia; 4 Department of Physical Therapy, Western University, London, Canada; 5 Department of Mechanical Engineering, The University of British Columbia, Vancouver, Canada; 6 Department of Radiology, The University of British Columbia Faculty of Medicine, Vancouver, Canada; 7 Department of Physical Therapy, The University of British Columbia, Vancouver, Canada; 8 Department of Orthopaedics, The University of British Columbia, Vancouver, Canada

**Keywords:** Osteoarthritis, Biomechanics, Knee, Orthopaedics, Evaluation

## Abstract

**Objective:**

To explore clinical characteristics in individuals with patellofemoral osteoarthritis (PFOA) compared to individually-matched asymptomatic controls. We also explored associations between functional performance and patient-reported symptoms with patellofemoral alignment.

**Methods:**

We assessed 15 individuals with PFOA and 15 individually-matched asymptomatic controls. In addition to physical examination and patient-reported questionnaires, we evaluated functional performance, lower extremity strength and range of motion, and patellar alignment (using MRI). We analysed group differences with Wilcoxon’s matched-pairs signed rank tests, and within-group associations with Spearman’s rank correlations.

**Results:**

We included 24 (80%) women with median (IQR) age of 56 (9) years and BMI of 22.8 (5.9) kg/m^2^. Individuals with PFOA reported lower quality of life (8/100 points lower EQ-5D-5L, p=0.02), and performed worse on two functional tests: repeated one-leg rises (median 16 fewer rises, p=0.04) and timed stair climb (1.2 s slower, p=0.03). There were no differences in strength tests performed or range of motion. Patellar proximal translation correlated with worse functional performance and worse patient-reported pain, function and self-efficacy, while lateral translation and lateral tilt correlated with worse knee-related quality of life (Spearman’s r ranging from 0.5 to 0.7).

**Conclusion:**

Functional performance was worse in individuals with PFOA, despite those individuals having no significant differences on lower extremity strength testing. Patellofemoral alignment was associated with worse functional performance as well as worse patient-reported outcomes, and it may represent one mechanism underpinning PFOA-related symptoms.

## INTRODUCTION

Radiographic patellofemoral osteoarthritis (OA) is present in approximately half of middle-aged and older individuals with knee pain or tibiofemoral OA^1^ and is associated with substantial pain^2–5^ loss of physical function^2^ and reduced quality of life^6^ Isolated patellofemoral OA commonly progresses to tibiofemoral (or whole knee) OA^7 8^ Thus, the patellofemoral joint is a compelling target for knee OA research and management.

Clinical features of patellofemoral pain, and knee OA of predominantly tibiofemoral involvement, are well known^9 10^ but less is known about patellofemoral OA, particularly in comparison to asymptomatic individuals. Almost exclusively, single studies have reported individuals with patellofemoral OA (compared to controls) have: reduced function (Timed Up and Go)^11^ weaker hip abduction and extension but comparable hip external rotation strength^12 13^ lower quadriceps strength^12^ smaller hip and quadriceps muscle volume^14 15^ worse patient-reported pain and function (Knee injury and Osteoarthritis Outcome Score, KOOS)^16^ altered pelvic and hip kinematics during gait (greater anterior and lateral pelvic tilt, greater hip adduction, lower hip extension)^17^ lower hip muscle activity during gait^18^ and reduced ankle dorsiflexion range of motion (ROM) but greater foot mobility with no difference in frontal plane knee alignment during single leg squats^19^ Several studies have reported greater lateral patellar tilt, lateral translation, and proximal translation in individuals with patellofemoral OA compared to asymptomatic controls^20–22^ With the exception of hip strength^12 13^ no two studies have yet to evaluate the same clinical features (physical exam, strength, ROM, functional performance), and no study has yet reported on a spectrum of clinical features together in one sample, or associations among such features. A more complete clinical picture of patellofemoral OA would inform clinical research aimed ultimately at primary and secondary knee OA prevention and treatment.

We aimed to describe and compare a wide range of clinical characteristics (physical examination, functional performance, strength, range of motion, and patellofemoral alignment) in individuals with patellofemoral OA to individually-matched controls. Our secondary aim was to explore associations between functional performance and patient-reported symptoms with patellofemoral alignment, as a possible mechanism underlying patellofemoral OA-related pain, loss of function, and quality of life.

## METHODS

### Participants

This cross-sectional pilot study reports clinical data acquired during a previous MRI (MRI) study in the same sample^20 23^ For the primary study, we recruited 15 individuals with patellofemoral OA and 15 individually-matched asymptomatic controls (see table 1 for eligibility criteria). Controls were matched to patellofemoral OA cases on age (within 5 years), sex, ethnicity, body mass index (BMI, within 5 kg/m^2^), and current physical activity level (low, moderate or high according to the International Physical Activity Questionnaire-Short, IPAQ-S)^24^ The study was approved by the University of British Columbia’s Clinical Research Ethics Board (ID H13-01993). All participants provided written, informed consent.

**Table 1:** Eligibility criteria

Patellofemoral OA (n=15)	Controls (n=15)
**Inclusion**	
Aged ≥40 years.	Aged ≥40 years.
Peri- or retro-patellar knee pain.	Asymptomatic for >1 year.
Pain aggravated by ≥1 activity that increases patellofemoral joint load (eg, squatting).	
Pain rated ≥3/10 on numeric pain rating scale.	
Pain on most days of previous month.	
At least doubtful patellofemoral osteophytes on skyline or lateral view radiographs, confirmed by a musculoskeletal radiologist (ie, KL Grade ≥1).	
**Exclusion**	
Pain elsewhere in the knee, hips, ankles, feet, or lumbar spine.	Any lower-limb symptoms in the past year.
Knee or hip arthroplasty, osteotomy, reconstruction, meniscectomy, fracture; history of major traumatic knee injury requiring non-weightbearing for ≥24 hour (eg, dislocation, complete ligament rupture).	Knee or hip arthroplasty, osteotomy, reconstruction, meniscectomy, fracture; history of major traumatic knee injury requiring non-weightbearing for ≥24 hour (eg, dislocation, complete ligament rupture).
BMI ≥35 kg/m^2^.	BMI ≥35 kg/m^2^.
Planned lower-limb surgery in the following 6 months.	Planned lower-limb surgery in the following 6 months.
Knee injections in the past 3 months.	Knee injections in the past 3 months.
Contraindications to imaging.	Contraindications to imaging.
Inability to understand written and spoken English.	Inability to understand written and spoken English.
Tibiofemoral joint OA severity of KL grade 3 or 4, or worse OA severity at the tibiofemoral joint than the patellofemoral joint.	

Controls were individually matched to patellofemoral OA cases on age, sex, ethnicity, BMI, and current physical activity level.

BMI, Body mass index; KL, Kellgren and Lawrence; OA, Osteoarthritis; OA classification criteria.

### Patient and public involvement

During protocol development and study design, we consulted with a variety of clinicians (physiotherapy, radiology, orthopaedists) through Grand Rounds and in-clinic presentations and discussions. Patients were not directly involved in study design.

### Clinical outcome measures

For participants with patellofemoral OA, we evaluated the painful knee, or most painful knee if pain was bilateral. For controls, we evaluated the knee with the same leg dominance as each matched case. All clinical tests are listed below, and are described in further detail in online supplemental table 1.

10.1136/bmjsem-2020-000877.supp1 Supplementary data

### Patient-reported outcome measures

Participants with patellofemoral OA completed four patient-reported outcome measures: KOOS (Symptoms, Pain, Activities of Daily Living, Sport and Recreation, Quality of Life and Patellofemoral subscales)^25 26^ Anterior Knee Pain Scale (AKPS)^27^ Tampa Scale of Kinesiophobia^28^ and Knee Self-Efficacy Scale (K-SES)^29^ Both groups completed the Euro-Quality of Life 5-Dimension, 5-Likert scale (EQ5D-5L)^30^ Participants reported whether they currently felt grinding in their knee^31^ had a history of anterior knee pain^31^ had a history of knee swelling^32^ current medication use, smoking history, and whether they had been diagnosed with OA in other joints in the body. These questions were asked to estimate comorbidity.

### Physical examination

We performed the following physical examination tests: swipe test (swelling)^33^; Beighton tests for generalised hypermobility^34^; tibiofemoral frontal plane alignment (inclinometer method)^35^; and Herrington’s test of mediolateral patellar positioning.^36^

### Performance-based tests

Participants performed: the 30-second chair stand test (maximum number of sit-to-stands in 30 s)^37^; repeated one leg rise test (maximum number of one legged sit-to-stands, to a metronome, to a maximum of 50 repetitions)^38^; and 12-step timed stair climb test (time to ascend and descend 12 steps)^37^

Participants performed five repetitions of a single leg squat to 45° knee flexion that was filmed (Sony HDR-CX580V digital HD video recorder) in the frontal plane to estimate 2D planar alignment at the pelvis, hip and knee^39 40^ We used the average of three successful trials (ie, the participant squatted to target and returned to full standing without the free foot touching the ground, typically repetitions two through four) to evaluate five alignment measures at the targeted angle (45° knee flexion). Knee frontal plane projection angle is a 2D measure of dynamic knee valgus (<180°=valgus). Pelvis level is a measure of the relative heights of the ipsilateral and contralateral anterior superior iliac spines (ASIS, >0°=contralateral hip hike). Hip adduction is a measure of the femur angle relative to the pelvis (<90°=hip adduction). Dynamic valgus index combines 2D hip adduction and knee valgus into a composite measure^39^ Sternal symmetry is a measure of how centred the trunk is positioned relative to the pelvis (<50%=sternal marker is closer to the ipsilateral ASIS along a line joining the two ASIS markers, meaning either the trunk is leaned towards the ipsilateral side or the contralateral pelvis is lower than the ipsilateral pelvis). All angles were measured using Motion Analysis Software version 9.9.0.1 (eHAB, Australia).

For ROM we evaluated the mean of two trials using a 12-inch handheld goniometer: knee extension and flexion; and hip extension, flexion, internal rotation and external rotation^41^ We also assessed ankle dorsiflexion with a knee-to-wall lunge test^42^ and normalised the distance from great toe to wall as a percentage of shank length.

We assessed peak isokinetic knee flexion and extension torques from trials of five repetitions at 60°/s and 180°/s^43^ (Biodex Multi-Joint System, Shirley, NY). We evaluated isometric torques (best of three trials, normalised to body weight) using a digital handheld dynamometer (Lafayette, IN) for hip extension, internal and external rotation, and abduction.^44^

### Imaging (patellofemoral alignment)

We measured both traditional supine two-dimensional (2D) and innovative three-dimensional (3D) patellofemoral alignment using MR imaging on two different scanners^20 23^ The supine 2D images allow comparison of our results with previous studies^22^ and the weightbearing 3D MR images represent a technical advance and more functionally-relevant position over the traditional 2D methods^20 23^ (see below).

To obtain 2D patellofemoral alignment, participants were scanned in a relaxed supine position in a 3T MR scanner (sagittal T1-weighted turbo spin echo sequence, Philips Achieve, Best, NL) using a commercially-available surface coil^3 20^ (online supplemental figure 1). This is the typical way that MR imaging is used to capture patellofemoral alignment in the literature^22^ We measured bisect offset, patellar tilt angle, and Insall-Salvati ratio (online supplemental figure 2), because these have been previously shown to differ in patellofemoral OA^20 22^

We captured images for 3D alignment in a vertically open-bore 0.5T MR scanner (sagittal plane, gradient echo sequence, ParaMed MROpen Genoa, Italy)^20 23^ Participants were positioned, fully weightbearing, in a single-leg squat with 30° of knee flexion—a position that commonly provokes pain in patients with anterior knee pain^45^ To quantify 3D alignment we (i) segmented bones on all image slices obtained in both the 3T and 0.5T scanners, (ii) created participant-specific anatomical surface models using 3T scanner images, (iii) registered the surface models to the bony outlines from the 0.5T images, and (iv) calculated alignment using assigned joint coordinate systems^20 23^ We measured patellar lateral translation, mediolateral tilt, and proximal translation because they are the weightbearing 3D equivalents of the 2D alignment measures included in this study. More detailed image acquisition protocols are described elsewhere^20 23^

### Statistical analyses

We described all participant demographics and examination findings separately by group (patellofemoral OA and control), as proportions or as median (IQR, IQR) due to the relatively small sample sizes in each group. To compare groups, we compared dichotomous outcomes as proportions using McNemar’s ᵡ² test. We compared continuous variables between the two groups using Wilcoxon’s matched-pairs signed rank tests. To calculate standardised effect sizes (d), we first confirmed normal distribution of the paired difference scores (Shapiro-Wilk tests), and then calculated d as mean/SD (SD) of the difference between matched pairs. This method is similar to Cohen’s d but accounts for individual matching. We considered a small effect size d ≥ |0.2|, moderate as d ≥ |0.5|, and large as d ≥ |0.8|^46^ and considered a moderate effect size to be potentially clinical relevant. Finally, we explored correlations between functional performance tests and patellofemoral alignment (both 2D and 3D) in all participants (n=30), and correlations between patient-reported outcomes and patellofemoral alignment in the patellofemoral OA group (n=15). For these analyses, we used Spearman’s rank correlation coefficients, and defined correlations as moderate with r ≥ |0.40|, strong r ≥ |0.6|, and very strong r ≥ |0.80|^47^ We considered a moderate correlation to be potentially clinical relevant.

Statistical significance was set at *p*≤0.05. All statistical analyses were completed using Stata Inter-cooled version 13.1 (StataCorp, Texas, USA).

## RESULTS

### Participant characteristics and patient-reported outcomes

The study sample included 24 (80%) women and 6 (20%) men, and median (IQR) age was 56 (9) years, BMI was 22.8 (5.9) kg/m^2^ (see table 2). Most of the sample reported being moderately or highly physically active in the previous week, with three participants (two with patellofemoral OA) reporting having been sedentary in the previous week. Twelve pairs were of white/European ethnicity, two pairs were of Chinese ethnicity, and one pair was of Indian and mixed Indian/Asian ethnicity. The entire sample reported being current non-smokers, and only two participants reported a smoking history, both controls (one 7.5 pack-year history, one 30 pack-year history, both quit approximately 15 years previously).

**Table 2:** Patient demographics

	Patellofemoral OA	Controls
Women, n (%)	12 (80%)	12 (80%)
Age, years	54 (10)	56 (8)
BMI, kg/m^2^	22.64 (7.95)	23.32 (5.93)
Physical activity past week, hours	5.30 (4.75)	7.50 (7.94)

All values are median (IQR) unless otherwise noted.

BMI, Body mass index; OA, Osteoarthritis.

The individuals with patellofemoral OA reported taking a total of 19 prescription medications, all unrelated to OA symptoms (eg, the most common prescription was synthroid): seven were taking no medications, five were taking one or two medications, one was taking three medications, and two were taking five medications. The control group reported taking a total of 10 prescription medications: seven were taking no medications, and the remaining eight were each taking one or two medications. Ten of the patellofemoral OA participants, and no controls, reported symptomatic OA in other joints, specifically eight (53%) in the contralateral knee, and three (20%) in the hands/fingers.

A higher proportion of participants with patellofemoral OA reported grinding in their knees, a history of anterior knee pain, and a history of dramatic swelling in the knee (table 3). Quality of life was significantly worse in the patellofemoral OA group, with large effect sizes (median 8 [IQR 20] points lower on the EQ-5D-5L VAS, |d|=1.0).

**Table 3:** Self-report scores and physical examination

	Patellofemoral OA	Controls	Paired difference	|d|	P
Reported crepitus n (%)	9 (60%)	0 (0%)			**<0.01**
History of anterior knee pain n (%)	15 (100%)	4 (26.7%)			**<0.01**
History of swelling n (%)	11 (73%)	0 (0%)			**<0.01**
Swipe test positive n (%)	8 (53%)	3 (20%)			0.06
Beighton score/9	0 (1)	1 (1)	−1 (1)	**0.5**	0.06
Tibiofemoral frontal plane alignment (°)	−1.0 (2.5)*	−0.5 (2)*	−1.5 (3.5)	**0.6**	**0.03**
Herrington test of mediolateral patellar positioning (M:L ratio)	1.19 (0.26)	1.07 (0.14)	0.11 (0.15)	**0.9**	**0.01**
EQ-5D-5L					
Index score	0.885 (0.044)	0.949 (0.00)	−0.06 (0.04)	**1.0**	**<0.01**
VAS	85 (10)	91 (10)	−8 (20)	**0.8**	**0.02**
KOOS					
Symptoms	71.4 (28.6)	–			–
Pain	75.0 (16.7)	–			–
Activities of Daily Living	83.8 (20.6)	–			–
Sport and Recreation	50.0 (45.0)	–			–
Quality of Life	56.3 (31.3)	–			–
Patellofemoral	56.8 (22.7)	–			–
AKPS	74 (22)	–			–
Tampa Scale for Kinesiophobia High (≥37) n (%)	34 (13) 7 (46.7%)	–			–
Knee Self-Efficacy Scale					
Daily Activities	84.3 (25.7)	–			–
Sport and Leisure	72 (28)	–			–
Physical Activities	51.7 (31.3)	–			–
Knee Function in the Future	53.3 (33.3)	–			–

*<0° is valgus alignment.

Bold indicates at least a moderate effect size between groups, or statistical significance.

All scores are median (IQR) unless otherwise stated.

AKPS, Anterior Knee Pain Scale (varies from zero, maximum problems, to 100, no problems); |d|, Standardised effect size; EQ-5D-5L, EuroQol Health Status Measure-5 dimension-5-likert: Index provides a Canadian-specific adjusted score combining all 5 dimensions (scores from zero [dead] to 1.000 [perfect health]), and VAS (visual analogue scale) is a single overall self-reported evaluation that varies from zero (dead) to 100 (perfect health); KOOS, Knee injury and Osteoarthritis Outcome Score (varies from zero, maximum problems, to 100, no problems); IQR, Interquartile range; OA, Osteoarthritis; M:L, Medial to lateral.

### Physical examination

A higher proportion of participants with patellofemoral OA had a positive swipe test (indicating knee swelling) than control participants (table 3). Beighton scores were low overall and did not differ significantly between groups. Clinical tibiofemoral frontal plane alignment was more valgus in the patellofemoral OA group, with a moderate effect size (1.5 [3.5]° more valgus, |d|=0.6). Herrington’s test of mediolateral patellar position showed patellae were more laterally displaced in the patellofemoral OA group, with a large effect size (0.1 [0.2] larger medial to lateral ratio, |d|=0.9).

### Performance-based tests

Two functional tests—repeated one leg rises and timed stair climb—differed significantly between groups (table 4). Specifically, participants with patellofemoral OA performed 16 (42) fewer repeated one-leg rises compared to controls, and they navigated stairs 1.2 (4.0) seconds more slowly (both were moderate effect sizes, |d|=0.6). All remaining functional performance tests, range of motion, and strength tests revealed no between-group differences. Frontal plane dynamic alignment during a single-leg squat also did not differ between groups at the trunk, pelvis, hip or knee. However, a higher proportion of individuals with patellofemoral OA (77% vs controls 38%) reported perceiving the task’s effort to be hard or markedly hard.

**Table 4:** Clinical performance. All values reported as median (IQR) unless otherwise stated

	Patellofemoral OA	Control	Paired difference	|d|	P
**Function**					
30 s chair stand test (n)	14 (2)	14 (6)	0 (6)	0.4	0.22
Repetitive one leg rises (n)	4 (34)	28 (34)	−16 (42)	**0.6**	**0.04**
Timed 12 stair climb (s)	10.1 (3.0)	8.9 (1.6)	1.2 (4.0)	**0.6**	**0.03**
**Range of motion**					
Ankle dorsiflexion/shank length (%)	24.0 (8.4)	30.6 (11.2)	−4.8 (17.3)	0.4	0.12
Knee hyperextension (°)	2.0 (6.0)	3.5 (4.0)	−2.5 (8.0)	0.4	0.14
Knee flexion (°)	137.5 (13.0)	135.0 (10.0)	0.0 (7.0)	0.1	0.73
Hip extension (°)	16.5 (8.0)	17.0 (8.0)	1.0 (5.0)	0.1	0.95
Hip flexion (°)	119.0 (24.0)	125.5 (16.0)	−1.0 (25.5)	0.3	0.39
Hip internal rotation (°)	39.0 (20.0)	36.5 (10.5)	−4.0 (19.0)	0.1	0.78
Hip external rotation (°)	44.0 (12.0)	48.5 (14.0)	−1.5 (19.5)	0.0	0.93
**Isokinetic strength**					
Knee extension peak torque, 60°/s (% normalised to BW)	145.6 (71.8)	175.2 (85.3)	−16.1 (161.3)	0.4	0.21
Knee extension peak torque, 180°/s (% normalised to BW)	105.1 (53.6)	127.9 (46.5)	−22.8 (93.3)	0.4	0.19
Knee flexion peak torque, 60°/s (% normalised to BW)	89.6 (28.2)	94.8 (32.2)	3.7 (41.1)	0.2	0.86
Knee flexion peak torque, 180°/s (% normalised to BW)	66.3 (24.3)	70.3 (31.8)	0.7 (28.5)	0.4	0.36
**Isometric strength**					
Hip extension (Nm/kg)	1.2 (0.5)	1.5 (0.4)	−0.3 (1.0)	0.2	0.64
Hip abduction (Nm/kg)	1.2 (0.4)	1.4 (0.7)	−0.3 (0.5)	0.4	0.22
Hip internal rotation (Nm/kg)	0.4 (0.3)	0.4 (0.4)	0.0 (0.5)	0.1	0.98
Hip external rotation (Nm/kg)	0.5 (0.2)	0.6 (0.4)	−0.1 (0.4)	0.2	0.47
**Single leg squats 2D angles**					
Knee abduction (°)*	174.3 (6.2)	173.9 (9.0)	−0.6 (12.6)	0.1	0.68
Pelvis level (°)†	1.2 (5.0)	1.9 (1.3)	−0.4 (4.8)	0.4	0.28
Hip adduction (°)‡	79.1 (7.1)	80.4 (7.7)	−0.5 (9.9)	0.1	0.75
Dynamic valgus index§	18.6 (14.0)	14.5 (14.4)	2.2 (22.5)	0.0	0.92
Sternal symmetry¶	41.2 (9.8)	43.5 (11.2)	−3.1 (17.5)	0.3	0.39
Perceived effort hard or very hard n (%)	10 (77%)	5 (38%)			**0.05**

*Angles <180°=valgus.

†Larger value=less contralateral hip drop.

‡Larger value=less hip adduction.

§Larger value=more combined hip adduction and knee valgus.

¶Horizontal distance of (ipsilateral anterior superior iliac spine, ASIS, to sternal marker) as a percentage of (contralateral ASIS to sternal marker), <50%=ipsilateral trunk lean or contralateral hipdrop.

BW, Body weight; |d|=Standardised effect size; IQR, IQR; OA, osteoarthritis.

Bold indicates at least a moderate effect size between groups, or statistical significance.

### Patellofemoral alignment

Bisect offset was 7 (27) % larger, indicating greater lateral displacement in individuals with patellofemoral OA compared to controls (moderate effect size, |d|=0.6, table 5). 3D mediolateral tilt revealed individuals with patellofemoral OA had 6 (12) ° more lateral patellar tilt than controls (moderate effect size, |d|=0.7). Effect sizes were also moderate for greater 2D patellar tilt angle (5 [12] ° more lateral tilt, |d|=0.5) and 3D proximal translation (8 [27] mm, |d|=0.5), but these did not reach statistical significance.

**Table 5:** Patellofemoral alignment on imaging

	Patellofemoral OA	Control	Paired difference	|d|	P
2D alignment, supine near full extension					
Insall-Salvati ratio	1.18 (0.15)	1.10 (0.31)	0.07 (0.47)	0.4	0.16
Bisect offset (%)	60.8 (26.2)	52.5 (6.5)	6.6 (27.5)	**0.6**	**0.05**
Patellar tilt angle (°)	14.3 (9.9)	9.5 (6.6)	5.5 (12.1)	**0.5**	0.08
3D alignment, single leg squat 30° flexion					
Proximal translation (mm)	18.5 (15.1)	11.9 (15.9)	7.5 (26.9)	**0.5**	0.11
Lateral translation (mm)	0.4 (9.8)	−1.8 (4.1)	2.3 (8.2)	0.4	0.19
Medial tilt (°)*	14.5 (6.3)	18.1 (10.0)	−5.9 (11.9)	**0.7**	**0.02**

*3D measure of tilt the value is reported as medial tilt, so a smaller value indicates greater lateral tilt.

All values reported as median (IQR) unless otherwise stated.

Bold indicates at least a moderate effect size between groups, or statistical significance.

|d|, Standardised effect size; IQR, Interquartile range; OA, Osteoarthritis.

The most frequent correlations between alignment and patient-reported outcomes or function was for 3D proximal patellar translation in weightbearing (table 6). Proximal translation was significantly associated with the following patient-reported outcomes: KOOS Pain (strong correlation, r=−0.63), AKPS (moderate correlation, r=−0.58), and K-SES Daily Activities subscale (strong correlation, r=−0.69). Proximal translation was also moderately correlated with the KOOS Activities of Daily Living (r=−0.49) and Patellofemoral (r=0.41) subscales, and the K-SES Sport & Leisure (r=−0.49) and Physical Activities (r=−0.48) subscales, but these did not reach statistical significance. Proximal translation was also the only measure that was significantly correlated with any functional performance tests: repeated one-leg rises (moderate correlation, r=−0.48) and timed stair climb (moderate correlation, r=0.48).

**Table 6:** Spearman’s rank correlations between patient-reported outcomes, function and key patellofemoral alignment measures

	3D measures (weightbearing)						2D measures (nonweightbearing)					
	Proximal translation		Lateral translation		Mediolateral tilt		Insall Salvati Ratio		Bisect offset		Patellar tilt angle	
	r	P	r	P	r	P	r	P	r	P	r	P
Patient-reported outcomes												
KOOS (n=15)†												
Symptoms	−0.24	0.39	**−0.45**	0.09	−0.34	0.22	0.23	0.41	−**0.43**	0.11	**−0.48**	0.07
Pain	**−0.63**	**0.01**	−0.31	0.27	−0.35	0.20	−0.03	0.93	−0.15	0.60	−0.11	0.68
Activities of Daily Living	**−0.49**	0.06	−0.22	0.41	−0.28	0.31	0.15	0.59	−0.03	0.93	−0.24	0.40
Sport and Recreation	−0.38	0.16	−0.39	0.16	**−0.40**	0.14	−0.11	0.70	−0.37	0.18	−0.36	0.18
Quality of Life	−0.20	0.47	**−0.63**	**0.01**	**−0.52**	**0.05**	−0.01	0.98	**−0.57**	**0.03**	**−0.65**	**<0.01**
Patellofemoral	**−0.41**	0.13	−0.28	0.31	−0.23	0.42	−0.08	0.77	−0.24	0.40	−0.30	0.27
AKPS (n=15)	**−0.58**	**0.02**	**−0.41**	0.13	**−0.50**	0.06	0.09	0.75	−0.30	0.28	−0.36	0.19
Tampa Scale for Kinesiophobia (n=15)	0.21	0.44	−0.01	0.98	0.08	0.77	0.19	0.49	−0.22	0.42	−0.26	0.36
Knee Self-Efficacy Scale (n=15)												
Daily Activities	**−0.69**	**<0.01**	−0.20	0.47	−0.34	0.22	−0.22	0.44	−0.10	0.72	0.01	0.98
Sport & Leisure	**−0.49**	0.06	0.03	0.91	−0.01	0.97	0.09	0.75	0.26	0.35	0.13	0.65
Physical Activities	**−0.48**	0.07	−0.39	0.15	**−0.45**	0.09	−0.06	0.83	−0.19	0.49	−0.16	0.57
Knee Function in the Future	−0.20	0.47	−0.20	0.48	−0.28	0.32	0.38	0.16	0.05	0.85	0.03	0.93
EQ-5D-5L (n=30)	−0.37	**0.04**	0.02	0.93	−0.31	0.10	**−0.42**	**0.02**	**−0.49**	**<0.01**	−0.35	0.06
Functional performance (n=30)												
30 s chair stand test	−0.19	0.31	−0.02	0.94	0.09	0.63	−0.04	0.85	0.16	0.39	0.08	0.68
Repetitive single leg rises	**−0.48**	**<0.01**	−0.07	0.72	−0.18	0.34	−0.32	0.09	−0.24	0.21	−0.22	0.24
Timed 12 stair climb	**−0.48**	**<0.01**	0.16	0.41	−0.15	0.43	−0.33	0.07	−0.03	0.88	0.06	0.77

†Only participants with patellofemoral osteoarthritis completed knee-related patient-reported outcomes (n=15); all remaining comparisons are for the full sample (n=30).

Items in bold indicate moderate correlation (r ≥0.40), or statistical significance (p≤0.05).

Correlation signs have all been standardised to reflect that a ‘–’ sign indicates that as the patella moves further into malalignment (ie, more proximally, more laterally, or more lateral tilt), the outcome of interest worsens.

AKPS, Anterior Knee Pain Scale; EQ-5D-5L, Euro-Quality of Life 5-Dimension, 5-Likert scale; KOOS, Knee injury and Osteoarthritis Outcome Score; PFOA, Patellofemoral osteoarthritis.

Of the remaining alignment measures, greater lateral translation and lateral patellar tilt (as evaluated by both 2D and 3D measures) were significantly correlated with worse KOOS Quality of Life (moderate to strong effect sizes, |r| ranging from 0.52–0.65). These alignment measures were also moderately correlated with other patient-reported outcomes, but no discernable pattern of association emerged. Insall-Salvati ratio was not correlated with any patient-reported outcomes or functional tests.

## DISCUSSION

In this pilot study, we found significant differences in patient-reported outcomes, functional performance tests, and patellofemoral alignment. Specifically, individuals with patellofemoral OA reported worse health-related quality of life, greater likelihood of crepitus and history of anterior knee pain and swelling, all of which are consistent with findings previously reported^6 31 32 48^ Individuals performed worse on two of three functional performance tests compared to controls. Patellofemoral alignment differed between groups on several measures: (i) 2D supine MR images (larger bisect offset, OA group), (ii) 3D weightbearing MR images (greater lateral tilt, OA group); and (iii) clinical measures (greater tibiofemoral valgus and lateral patellar displacement, OA group). All significant effect sizes (d) were at least moderate, suggesting possible clinical importance. These results can be used to inform future hypotheses and point to associations that may be clinically meaningful, thus guiding selection of outcome measures in future studies. These results also provide estimates for sample sizes calculations for future studies. For example, based on our stair climb test results, a sample size of 36 per group would be needed to achieve power of 0.80 and α of 0.05 to detect between-group differences.

We did not detect muscle weakness in individuals with patellofemoral OA, despite reduced functional performance. This differs from a previous study where, compared to healthy controls, eight individuals with patellofemoral OA had lower quadriceps strength and lower hip abduction and extension strength, but similar external rotation strength^12^ and another study where 15 individuals with patellofemoral OA had lower hip abduction strength but similar external rotation strength^13^ A possible explanation is that we matched controls on physical activity, whereas previous studies did not. Their findings may thus reflect deconditioning secondary to OA-related reductions in physical activity.

While muscle strength is one possible factor explaining functional performance, other factors may include: (i) pain avoidance or compensatory strategies (similar to antalgic gait patterns); (ii) psychological reluctance or lack of confidence to perform functionally demanding tasks; or (iii) differences in knee geometry. This latter point is supported by findings in our study that 3D patellar proximal translation correlated with worse functional performance, and worse patient-reported pain, function and self-efficacy in the patellofemoral OA group. A higher positioned patella could impair functional performance because it reduces contact area (resulting in greater joint contact forces and possibly more pain), or because the change in alignment negatively impacts the effective moment arm, resulting in decreased force generation about the knee.^49^

Interestingly, worse clinical outcomes were associated with 3D proximal patellar translation but not 2D Insall Salvati ratio, which is designed to identify patella alta^50^ The Insall Salvati ratio may be less sensitive because it is a 2D proxy measure for a more complex, 3D patellar position. Alternatively, it may be because the 2D measure was of a relaxed knee. In weightbearing, patellofemoral joint contact forces are higher, and active quadriceps may cause further proximal translation, both possibly contributing to worse symptoms.

Increased patellar lateral translation and lateral tilt (in both 2D and 3D) were related to worse patient-reported knee-related quality of life. This extends previous findings of lateral translation 1 year after anterior cruciate ligament reconstruction predicting reduced KOOS Quality of Life 5-years post-surgery^51^ These findings warrant further investigation, particularly because alignment may be modifiable through treatments such as taping and bracing.^52–54^

The only functional test that did not differ between groups was the timed chair stand, a recommended test for whole-knee OA^37^ This task is less demanding than single leg rises and stair climbing. Our results suggest this test may have a ceiling effect in physically active individuals with predominantly patellofemoral OA. More demanding tasks such as the repeated one-leg rise, in addition to recommended core outcome sets, may improve sensitivity for detecting early functional decline when evaluating individuals with patellofemoral OA, who may represent an earlier stage of knee OA.

The primary limitation to this study is its small sample size. While individual-matching-reduced confounding, this was nonetheless a pilot study that is underpowered for confirmatory analyses. We intentionally evaluated a wide range of clinical characteristics, which is a strength of the study given how little is known about patellofemoral OA. However, on account of this being an exploratory study design, and thus hypothesis-generating, we did not adjust for multiple testing^55^ Doing so can reduce the likelihood of spurious findings (Type I errors), but this occurs at the expense of increasing the likelihood of false negatives (Type II errors)^55^ The results of this pilot study should therefore be considered within this context, and in addition to p-values, effect sizes and direction of effects should be considered to identify potentially interesting findings that can be confirmed in future larger studies. A third limitation is that patient-reported physical activity is prone to bias^56^ which may have introduced error into our matching methods. Finally, while our methods for evaluating hip strength have adequate reliability^44^ we are unable to assess for specific hip muscle weakness. Having said this, previous studies found that all hip abductor muscles had smaller volume in patellofemoral OA compared to controls, suggesting that any hip weakness may be generalised rather than selective.^14^

## CONCLUSIONS

Our study identified a variety of clinical features that differed in individuals with patellofemoral OA compared to individually-matched controls. Most notably, functional performance was worse in individuals with patellofemoral OA, despite no significant differences in strength testing. Patellofemoral malalignment was associated with worse functional performance and self-reported pain, function and quality of life. Alignment is thus a possible mechanism underpinning patellofemoral OA symptoms, and because alignment is modifiable^52–54^ this warrants further investigation. Overall, our findings inform future studies ultimately aimed at improving clinical management of patellofemoral OA.

**What are the new findings**

- We explored a broad spectrum of clinical features within a sample of individuals with PFOA compared to individually-matched controls, including: physical examination, patient-reported questionnaires, functional performance, strength, range of motion, and patellofemoral joint alignment.
- Functional performance was worse in individuals with PFOA despite no differences in strength testing or range of motion, compared to controls.
- Patellofemoral alignment differed between individuals with PFOA and controls, and also correlated with functional performance and patient-reported pain, function, quality of life, and knee-related self-efficacy.
