# Supplementary material for: Clinical findings in patellofemoral osteoarthritis compared to individually-matched controls: a pilot study
Source: BMJ Open Sport Exerc Med. 2020 Dec 9;6(1):e000877. doi: 10.1136/bmjsem-2020-000877 (PMC8323464; doi:10.1136/bmjsem-2020-000877)
Supplement: Supplementary data [file bmjsem-2020-000877supp001.pdf]

Supplementary Table 1: Description of all clinical tests

Test	Protocol description
**Tibiofemoral frontal plane alignment**	• Palpate & mark tibial tuberosity & midpoint over the talus neck<br>• Ask participant to stand on footprint map with foot at 10° external rotation, feet shoulder width, looking forward, 50% weightbearing<br>• Place callipers of inclinometer in alignment with the the two landmarks<br>• Record varus/valgus direction in degrees
**Herrington test**	• Participant supine on plinth, knee positioned and supported in 20° of knee flexion (to place the patella within the trochlea groove)<br>• With knee in position, place a piece of 1" Leukotape (or similar) across the knee joint, and mark the medial and lateral epicondyles of the femur and mid-point of the patella. Be sure to make note of medial and lateral end of tape<br>• Repeat 3 times, attaching tape to this document for measuring later
**30 second chair stand test**	• **Shoes on**, middle of chair, feet ~ shoulder width apart and slightly behind knees with feet flat on floor, arms crossed on chest<br>• Instructions *"stand up keeping arms across chest, and ensure you* stand completely up so hips and knees are fully extended; then sit completely back down, so that the bottom fully touches the seat, *as many times as possible in 30 seconds,"*<br>• 1-2 practice repetitions for technique<br>• One 30-second test trial<br>• Record number of correctly performed full stands (if more than ½ of way up at end of the test, counted as a full stand)
**Repetitive single leg rise test**	• **Shoes on**, seated on edge of plinth, foot placed with heel 10 cm forward from a plumb line at edge of plinth, other leg held at side of body, arms across chest.<br>• Height of plinth adjusted so knee angle is 90°<br>• Instructions: *"keep back of heel on marker, stand as many times as possible on one leg keeping arms across chest, in time with the* ***metronome (45bpm)****. If you get to 50 we will stop. Control yourself on the descent"*<br>• 2-3 practice tests

	• If nonweightbearing leg touches ground, do not count that rise; if touches ground 3 times during test, record number of rises to that point only and stop test
**Timed 12 stair climb**	• Participant wears usual footwear<br>• Instructions *“climb up then down the 2 small flights of stairs as quickly as possible but safely”*<br>• 1-2 practice trials for technique and to confirm safety<br>• Trial: record time to ascend and descend 12 steps - up 2 flights of 6 steps then descend<br>• (both feet touch down at bottom of landing to end timer)<br>• No use of hand-rails or gait aids<br>• Height of step: 18 cm<br>• Depth of step: 32 cm
**Ankle dorsiflexion range of motion (ROM)**	• Participant stands in front of a wall with horizontal line bisecting calcaneus and second toe<br>• Participant instructed to lunge forward to touch knee to wall<br>• Move foot back gradually until can just touch wall while maintaining heel on floor<br>• Measure distance between tip of great toe and wall<br>• Measure shank length from lateral knee joint line to lateral malleolus
**Knee hyperextension ROM**	• Participant supine on plinth, towel under Achilles tendon<br>• Measure ROM using 12” goniometer – axis over lateral epicondyle of femur; stationary arm towards greater trochanter; movable arm toward lateral malleolus
**Knee flexion ROM**	• Participant supine on plinth, slides heel towards buttocks without lifting heel off plinth<br>• Measure ROM using 12” goniometer – axis over lateral epicondyle of femur; stationary arm towards greater trochanter; movable arm toward lateral malleolus
**Hip extension ROM**	• Participant prone, feet off end of plinth, chin resting on hands<br>• Belt across pelvis<br>• Measure ROM using 12” goniometer – axis over greater trochanter; stationary arm parallel to mid-axillary line of trunk; movable arm toward lateral epicondyle of femur<br>• Knee maintained in extension
**Hip flexion ROM**	• Participant supine on plinth, bends knee and draws knee towards chest without using hands<br>• Measure ROM using 12” goniometer – axis over greater trochanter; stationary arm parallel to mid-axillary line of trunk; movable arm toward lateral epicondyle of femur
**Hip internal rotation ROM**	• Participant prone on plinth, test leg in 90° knee flexion<br>• Belt across pelvis

	• Inclinometer held to outside of shin 5 cm proximal to lateral malleolus, starting angle is 0°.<br>• Instructions: *"let leg drop out to side as far as possible"*<br>• Record angle without overpressure
**Hip external rotation ROM**	• Participant prone on plinth, test leg in 90° knee flexion, non-test leg in slight abduction for clearance<br>• Belt across pelvis<br>• Inclinometer held to inside of shin 5 cm proximal to medial malleolus, starting angle is 0°.<br>• Instructions: "*let leg drop in to centre as far as possible*"<br>• Record angle without overpressure
**Isokinetic knee extension / flexion strength (Biodex)**	• Participant seated in Biodex, back rest at 70-85°<br>• Two speeds: 60° and 180° per second<br>• 1-2 sub-maximal practice trials (~50% effort)<br>• One max test trial at each speed, 5 repetitions<br>• ≥ 30 second rest between each test series<br>• Verbal encouragement "*Go! Harder!*"<br>• If > 15% coefficient of variance, repeat test
**Hip abduction strength**	• Isometric 'make' test, 3-5 second hold<br>• Participant supine, belt across contra-lateral thigh<br>• Index leg resting in hip neutral (0°)<br>• Force plate of handheld dynamometer positioned 5 cm above lateral malleolus<br>• Instructions *"keep arms folded, heel on the bed and push leg out to side against force plate as hard as possible"*<br>• 1-2 sub-maximal practice trials<br>• Verbal encouragement "Go! Go! Go!"<br>• ≥ 5 second rest between each repetition<br>• If > 10% variability, additional trial is performed<br>• Measure lever arm length: greater trochanter to dynamometer marker (5 cm proximal to lateral malleolus)
**Hip extension strength**	• Isometric 'make' test, 3-5 second hold<br>• Positioned in prone, belt across contra-lateral thigh, chin resting on hands, feet off the end of the bed<br>• Index leg at 90º knee flexion<br>• Force plate over centre of participant's heel<br>• Instructions "*push foot straight up to ceiling*"

	• Therapist matches force once knee leaves surface of plinth.<br>• 1-2 sub-maximal practice trials<br>• Verbal encouragement “Go! Go! Go!”<br>• ≥ 5 second rest between each repetition<br>• If > 10% variability, additional trial is performed<br>• Measure lever arm length: inferior greater trochanter to lateral knee joint line
**Hip internal rotation strength**	• Isometric ‘make’ test, 3-5 second hold<br>• Positioned in prone, belt across contra-lateral thigh, index leg resting in hip neutral (0°), knee at 90°<br>• Force plate positioned 5 cm above lateral malleolus<br>• Instructions *“turn shin outwards as hard as possible, keeping both knees together”*<br>• 1-2 sub-maximal practice trials<br>• Verbal encouragement “Go! Go! Go!”<br>• ≥ 5 second rest between each repetition<br>• If > 10% variability, additional trial is performed<br>• Measure lever arm length: knee joint line to dynamometer marker (5 cm proximal to lateral malleolus)
**Hip external rotation strength**	• Isometric ‘make’ test, 3-5 second hold<br>• Positioned in prone, belt across contra-lateral thigh, index leg resting in hip neutral (0°), knee at 90°<br>• Force plate 5 cm proximal to medial malleolus<br>• Instructions *“turn shin inwards towards the centre as hard as possible”*<br>• 1-2 sub-maximal practice trials<br>• Verbal encouragement “Go! Go! Go!”<br>• ≥ 5 second rest between each repetition<br>• If > 10% variability, additional trial is performed<br>• Measure lever arm length: knee joint line to dynamometer marker (5 cm proximal to medial malleolus)
**Single leg squats**	• Video camera 3 metres from participant, at height of lowest tripod setting (~65 cm)<br>• Adjust height of plinth behind participant to provide squat depth target to 45° knee flexion, using 12” goniometer<br>• Place reflective markers: midpoint of femoral condyles, bilateral ASIS, proximal thigh along line from ASIS to knee marker, midpoint of ankle malleoli, manubrium<br>• Participant stands, **shoes off**, on foot map with arms across chest<br>• free leg held in front of squat leg

	• metronome 60 bpm, 2 ‘ticks’ each direction<br>• single leg squat to 45° knee flexion (plinth behind participant – buttock touch plinth as target to reproduce angle)<br>• 2-3 practice trials<br>• Repeat 5 times<br>• On test completion, participants were asked “*How easy was it to perform the task?*”. Response options were on a 5-point Likert scale ranging from ‘markedly easy’ to ‘markedly hard’

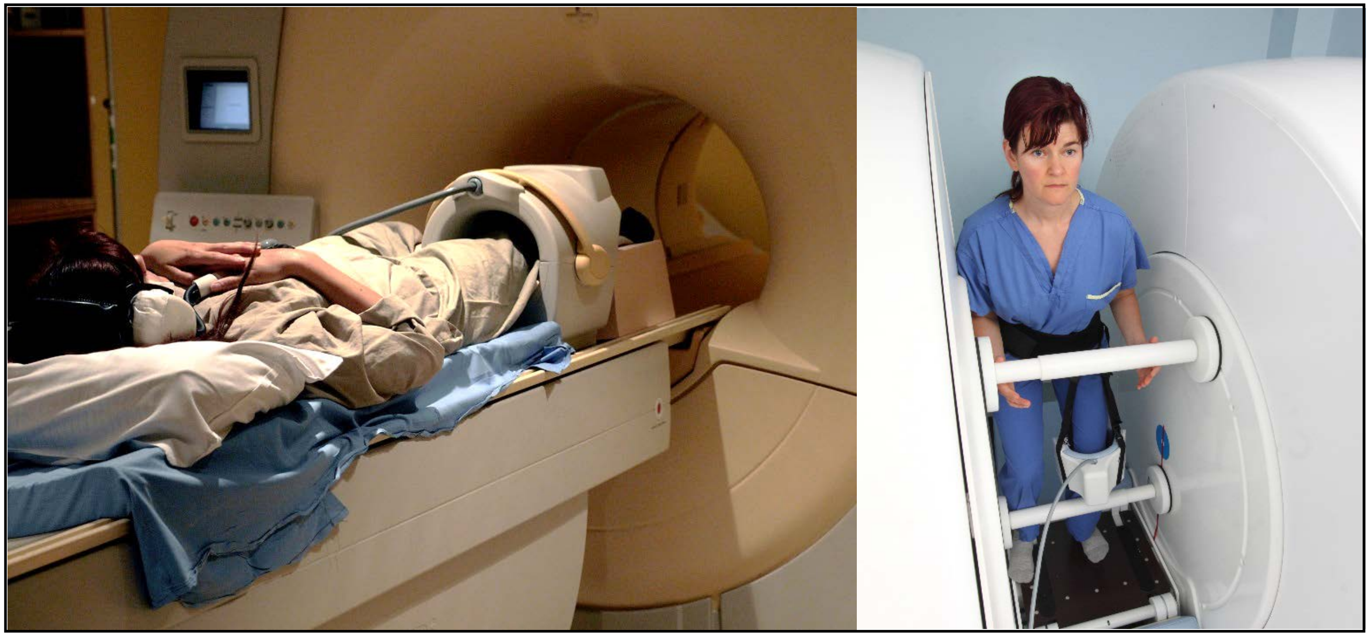

Supplementary Figure 1: Participants were positioned in supine for 2D (left) and upright for 3D (right) alignment measures. Note image on right shows standing two-legged, but in the present study participants stood on one leg only. *Right-hand image from Journal of Magnetic Resonance Imaging, Macri EM et al., "Patellofemoral and tibiofemoral alignment in a fully weight-bearing upright MR: Implementation and repeatability" Published Online First: Doi:10.1002/jmri.25823, 2017, with permission from John Wiley and Sons.*

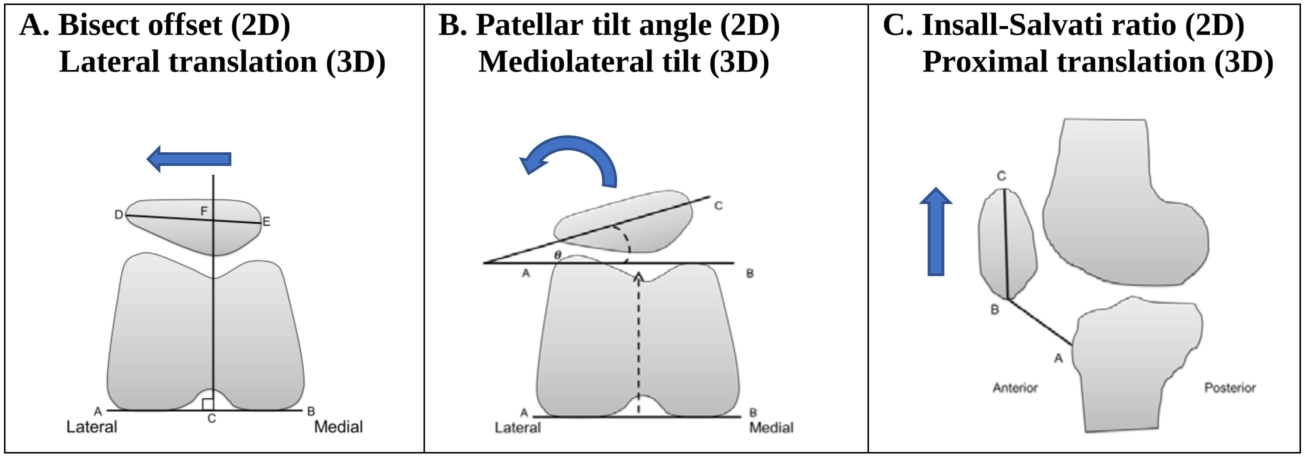

Supplementary Figure 2: Description of alignment measures

A. 2D bisect offset is the percentage (%) of the widest line across the patella that lies lateral to a line bisecting the deepest part of the trochlea (arrow indicates direction of 3D lateral translation of patella relative to femur, in mm). **B.** 2D patellar tilt angle: Angle (θ) between posterior condylar line and line across widest part of patella (arrow indicates direction of 3D lateral tilt of patella relative to femur, in degrees). **C.** 2D Insall-Salvati Ratio is the ratio of patella tendon length to the patellar height (arrow indicates direction of 3D proximal translation of patella relative to femur, in mm)
